# Polio in Afghanistan: The Current Situation amid COVID-19

**DOI:** 10.4269/ajtmh.20-1010

**Published:** 2020-08-27

**Authors:** Attaullah Ahmadi, Mohammad Yasir Essar, Xu Lin, Yusuff Adebayo Adebisi, Don Eliseo Lucero-Prisno

**Affiliations:** 1 Kabul University of Medical Sciences, Kabul, Afghanistan;; 2 Department of Thoracic Surgery, The First Affiliated Hospital, College of Medicine, Zhejiang University, Hangzhou, People’s Republic of China;; 3 Faculty of Pharmacy, University of Ibadan, Ibadan, Nigeria;; 4 Department of Global Health and Development, London School of Hygiene and Tropical Medicine, London, United Kingdom;; 5 Faculty of Management and Development Studies, University of the Philippines (Open University), Los Banos, Philippines

## Abstract

Polio is a deadly viral disease that has been paralyzing many children in Afghanistan. Despite fundamental efforts, primarily vaccination, to reduce the number of cases in Afghanistan, there are still many children who are deprived of the vaccine every year. Afghanistan is one of the two remaining countries endemic for polio, and the country has undergone various challenges that have hampered the eradication of this disease. The underlying challenges include inaccessibility of unsecured areas, illiteracy, refusal, and, most recently, COVID-19. The country is in the midst of a battle against COVID-19, and polio has almost entirely been neglected. Sadly, polio cases are increasing in the country, particularly in polio-free provinces. After an initial lockdown, many businesses have been allowed to resume, but the mass polio vaccination campaign has not restarted. New cases of polio will surge if endemic regions remain unvaccinated or inaccessible. To curb the further spread of polio, Afghanistan needs to resume nationwide house-to-house vaccination as restrictions due to COVID-19 are loosened.

## INTRODUCTION

Polio (poliomyelitis) is caused by a highly contagious virus that attacks the nervous system and can lead to paralysis and death. The virus infects unimmunized children through ingestion of contaminated water and food, and it spreads back to the environment through their feces. Interestingly, 90% of infected individuals do not exhibit any symptoms and remain undetected yet are carriers of the virus, which can spread to others. Thus, mass immunity is required to avoid polio outbreaks.^1^ This goal can only be fulfilled through mass polio vaccination campaigns, which have proven to be very effective, resulting in a > 99% decrease in the total number of worldwide cases, from an estimated 350,000 in 1988 to 33 reported in 2018.^2^ Moreover, the number of endemic countries has decreased from 125 in 1988^2^ to two—Afghanistan and Pakistan—in 2020.^1^

The Global Polio Eradication Initiative (GPEI) is a public–private partnership comprising six organizations—the WHO, the U.S. CDC, UNICEF, Rotary International, the Bill & Melinda Gates Foundation, and the Global Alliance for Vaccines and Immunizations. The GPEI implements the program, aiming to eradicate polio from the world. The GPEI decided to halt polio vaccination until the second half of 2020 in a move to use polio resources in the fight against COVID-19 and to prevent its spread among the vaccinators.^3^ Subsequently, there are concerns that new polio cases will soar and that the disease will enter polio-free regions, as happened in Nigeria in 2003, when vaccination cessation resulted in spread to 16 previously polio-free countries, including Angola, Chad, and the Democratic Republic of Congo.^4^ According to the WHO, if polio is not eradicated in Afghanistan and Pakistan, it could spread across the world, with 200,000 new cases every year, within 10 years.^2^

**Figure 1.**
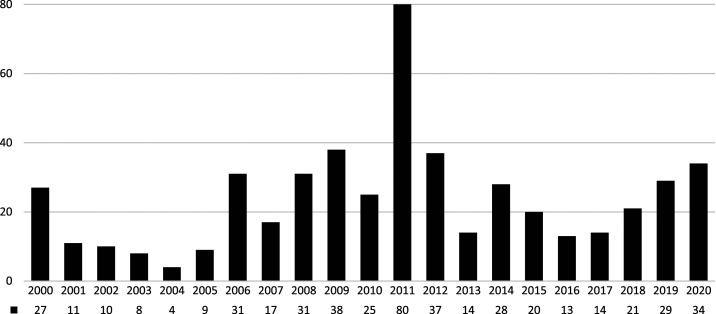
Wild polio cases in Afghanistan. Numbers for 2020 are through August 1, 2020. Source: Global Polio Eradication Initiative. Wild polio cases in Afghanistan. Cases per year are shown.

New cases of polio have considerably decreased in Afghanistan. The disease does not exist in 96% of the country. However, the country still records a considerable number of new cases every year. From 1997 to 2013, the annual number of confirmed cases fluctuated between 4 in 2004 and 80 in 2011 (Figure 1).^5,6^ In 2018, 21 confirmed cases of polio were recorded. Despite the launching of door-to-door vaccination programs in 2019, total confirmed cases reached 29 in that year. In 2020, 34 confirmed cases have been reported as of August 1, 2020.^6^

## EFFORTS

Even as house-to-house immunization campaigns were paused, the work of polio workers continued in some parts of the country. Under UNICEF supervision, polio social mobilizers ran the Immunization Communication Network, providing mother and child health referral services and assisting families to follow up their children’s health records. These mobilizers are from the community. Thus, they know the families well and can trace their immunization routines. Since the start of the pandemic in the country, the polio mobilizers have facilitated tracking the routine immunization schedules of more than 37,000 children in eastern and southern regions of Afghanistan and have provided them with referral vouchers to go to vaccination centers. Polio mobilizers are also cautioning people about the COVID-19 outbreak and how infection can be prevented through basic public health measures.^7^

Several complementary approaches have been implemented to mitigate polio risks. One is to raise awareness regarding the importance of immunization through regional and local media and influential religious leaders. UNICEF coordinates other related services regarding health, water, sanitation, and nutrition to accompany immunization services. It coordinates with Pakistan to place vaccination centers along borders between the two countries to vaccinate moving populations.^7^

## CHALLENGES

Polio has been challenging the fragile healthcare system of Afghanistan for a long time. The country has been constantly fighting to fully eliminate polio, but the threads of problems have made it complicated.

At least 95% of children younger than 5 years should be vaccinated to eradicate the virus.^8^ However, the ongoing conflict in the country has been changing the trajectory of polio elimination. With prolonged insecurity, the program is not implemented fully, and many children younger than 5 years are missing vaccinations, primarily in insecure provinces.^9^ More than 840,000 children missed vaccination opportunities in 2018 in six provinces, mainly in inaccessible southern and eastern regions.^10^ According to the United Nations Assistance Mission in Afghanistan, of the 29 cases reported in 2019, 23 were in inaccessible regions.^11^ In addition, in some cases, GPEI health workers have been targeted and killed while on assignment.^12^ Although a peace deal between the United States and the Taliban has brought some level of hope for appropriate health services for Afghans, including polio vaccination, an intense battle between anti-government and pro-government forces remains in progress.

Illiteracy, misinformation, and parental refusal are other barriers to polio eradication. Almost 600,000 Afghan children miss vaccination because of the refusal of their parents because of mistrust and the belief that it is prohibited in Islam.^13^ Some people think it is a Western conspiracy aiming to sterilize Muslim children.^14^ Besides that, huge population movement across the 2,700-km border between Afghanistan and Pakistan hinders vaccination efforts.^13^

Following GPEI’s recommendation to pause door-to-door polio vaccination, the biggest challenge in the battle against polio was in Afghanistan. National immunization days aim to cover around 10 million children against polio,^7^ and 10 nationwide polio vaccination programs are usually conducted every year. However, in 2020, only two programs were conducted before the outbreak of COVID-19 in the country.^14^ In addition, vaccine stocks ran precariously low as supply chains were disrupted by travel bans. Many health facilities where children were normally vaccinated were closed. The current pandemic is reigniting troubles in some of the fragile places where access to basic health and hygiene is limited.^7^ COVID-19 has contributed in pushing polio beyond its endemic regions of the south and southeast, and now, it threatens everyone throughout the country. Polio cases have recently emerged in three provinces, Balkh, Herat, and Badakhshan, which had not reported any cases in the past 5 years.^14^ On May 21, 2020, the government of Afghanistan ratified an initial plan for loosening restrictions and allowing some businesses, including money exchange markets and shops, to reopen.^15^ However, this plan did not include restarting nationwide polio vaccination, and as of August 1, 2020, the program remains suspended.

## CONCLUSION

The fight against polio, which has been waged in Afghanistan for a long time, has played a substantial role in controlling the disease. There are still insecure places in the country which present new cases every year. With COVID-19 dominating priorities, the country’s existing health facilities and resources have been reallocated to the fight against the pandemic, resulting in an increase in polio cases, particularly in previously polio-free provinces. New cases of polio will surge if endemic regions remain unvaccinated or inaccessible. For Afghanistan to curb the further spread of polio, it needs to resume nationwide vaccination within the policy of gradual relaxation of pandemic restrictions, rather than shifting all resources to COVID-19.
